# Supplementary material for: MMTF—An efficient file format for the transmission, visualization, and analysis of macromolecular structures
Source: PLoS Comput Biol. 2017 Jun 2;13(6):e1005575. doi: 10.1371/journal.pcbi.1005575 (PMC5473584; doi:10.1371/journal.pcbi.1005575)
Supplement: S1 Appendix — Links to software repositories, documentation, and benchmarks. (PDF) [file pcbi.1005575.s005.pdf]

**S1 Appendix. Software and Documentation.**

The MMTF website (http://mmtf.rcsb.org) contains links to the specification, software repositories, installation instructions, API documentation, and FAQ.

The code to run the benchmarks is available at:
https://github.com/rcsb/mmtf-java-benchmark

https://github.com/rcsb/mmtf-python-benchmark
